# Investigation of microbiological non-compliance of endoscopic final rinse water associated with opportunistic premise plumbing pathogens contamination in connecting tube

**DOI:** 10.1038/s41598-026-46256-9

**Published:** 2026-03-28

**Authors:** Miao Liu, Qingqing Zu, Linwen Zheng, Lirong Wen, Shanshan Li, Yuli Nie, Fenglin Chen, Qiaoyu Zhang

**Affiliations:** 1 Department of Gastrointestinal Endoscopy Nursing, Fujian Medical University Union Hospital, Fuzhou, 350001 Fujian Province China; 2 Fujian Clinical Research Center for Digestive System Tumors and Upper Gastrointestinal Diseases, Fuzhou, 350001 Fujian Province China; 3 Department of Nosocomial Infection Control, Fujian Medical University Union Hospital, Fuzhou, 350001 Fujian Province China; 4 Department of Gastroenterology, Fujian Medical University Union Hospital, Fuzhou, 350001 Fujian Province China

**Keywords:** OPPPs, Connecting tube, Contamination, Environmental sciences, Health care, Microbiology

## Abstract

Non-compliant final rinse water (microbial culture results exceeding 10 cfu/100 mL) is an under-recognized risk factor for failure of flexible gastrointestinal (GI) endoscope reprocessing, since it is an important step following disinfection during the reprocessing of flexible endoscopes. Opportunistic premise plumbing pathogens (OPPPs) may colonize terminal segments of the water distribution system and compromise rinse water quality. This study investigated the cause of recurrent final rinse water non-compliance in a GI endoscopy unit and evaluated an engineering intervention to prevent it. During August 2024, samples were collected from 34 reprocessed flexible gastrointestinal endoscopes (102 post-reprocessing samples) and various points within the water delivery system, including purified water and final rinse water from automated endoscope reprocessors and manual washing tanks. All samples were subsequently cultured for microbial analysis. After contaminated connecting tubes were identified as a potential OPPPs reservoir, 10 tubes between the circulation loop and the reprocessing equipment were replaced, and surveillance cultures were repeated at the same sampling sites. Initial assessments revealed low compliance rates of 32 water samples (37.5%, 12/32), according to infection control standards (≤ 10 cfu/100 mL). Water samples collected before connecting tubes showed 100% compliance (12/12 samples). Water collected through connecting tubes and final rinse taps all showed 0% compliance (0/10 samples), except for the water sample from AER 6, which had a bacterial count of 75 cfu/100mL, all other samples contained too numerous bacteria and could not be counted. The dominant bacteria is *Sphingomonas paucimobilis*, which was found in 14 samples, with a composition ratio is 43.75% (14/32). Others are *Methylobacterium oryzae* (8/32, 25%), *Chryseobacterium indologenes* (6/32, 18.75%), and *Herbaspirillum huttiense* (2/32, 6.25%). After replacing the connecting tubes, the compliance rate of water samples developed significantly to 100% (32/32) (*P* = 6.9 × 10^− 8^). The primary reason for non-compliance of final rinse water is the contamination of connecting tube by the OPPPs. These findings emphasize the need for shortening the connecting tube length and thorough cleaning and disinfecting of connecting tube between circulating tube and endoscope reprocessor, which are generally neglected in routine maintenance.

## Introduction

The cleaning, disinfection, and sterilization of flexible gastrointestinal (GI) endoscopes are critical aspects that demand significant attention due to their direct impact on patient safety and infection control^1^. The reprocessing of flexible endoscopes involves multiple disciplines and areas, including architectural layout, facilities and equipment, clinical diagnosis and treatment, cleaning and disinfection techniques, personnel evaluation, and quality surveillance^2,3^. The complete reprocessing cycle comprises multiple steps, each of which must be controlled to ensure that endoscopes are safe for reuse^1,3^. As the final flushing process after disinfection, the quality of the flushing water used in final rinse is crucial in determining whether the entire disinfection process is ultimately qualified and in assessing the risk of subsequent hospital-acquired infections^4^. OPPPs are opportunistic pathogens native to aquatic environments that proliferate in premise plumbing under conditions like stagnation, posing a contamination risk to medical water and equipment in healthcare settings^5,6^. The disinfection guidelines recommended by the “Regulation for Cleaning and Disinfection Technique of Flexible Endoscope” (WS507-2016, the Health Industry of the People’s Republic of China) recommends strictly using purified water or sterile water for the terminal rinsing of flexible endoscopes and ensuring that the total bacterial count of the purified water is ≤ 10 cfu(colony forming units)/100 mL.

Regular biological surveillance enables the detection of microbial contamination, providing a foundation for implementing targeted improvement measures. These efforts can significantly enhance the safety and reliability of endoscopic diagnosis and treatment procedures. In August 2024, during our quarterly routine biological surveillance of the Digestive Endoscopy Center of the Fujian Medical University union hospital (a tertiary-care teaching hospital with 3,500 beds located in southeastern China), the bacterial count in final rinse water exceeded the acceptable limit. However, the disinfection guidelines recommended by the “Regulation for Cleaning and Disinfection Technique of Flexible Endoscope” failed to completely eliminate the infection of the final rinse water. This study was performed to identify the possible reason for the non-compliance of endoscopic final rinse water.

## Method

### Setting

Our hospital is a tertiary-care teaching hospital with 3,500-bed located in southeastern China. There are 3 endoscope centers including the Interventional Treatment Center of Respiratory Diseases, Center for Digestive Endoscopy, and the Center for Ear, Nose, and Throat Endoscopy. More than 60,000 digestive endoscopy procedures are performed annually at the Center for Digestive Endoscopy.

**Fig. 1:**
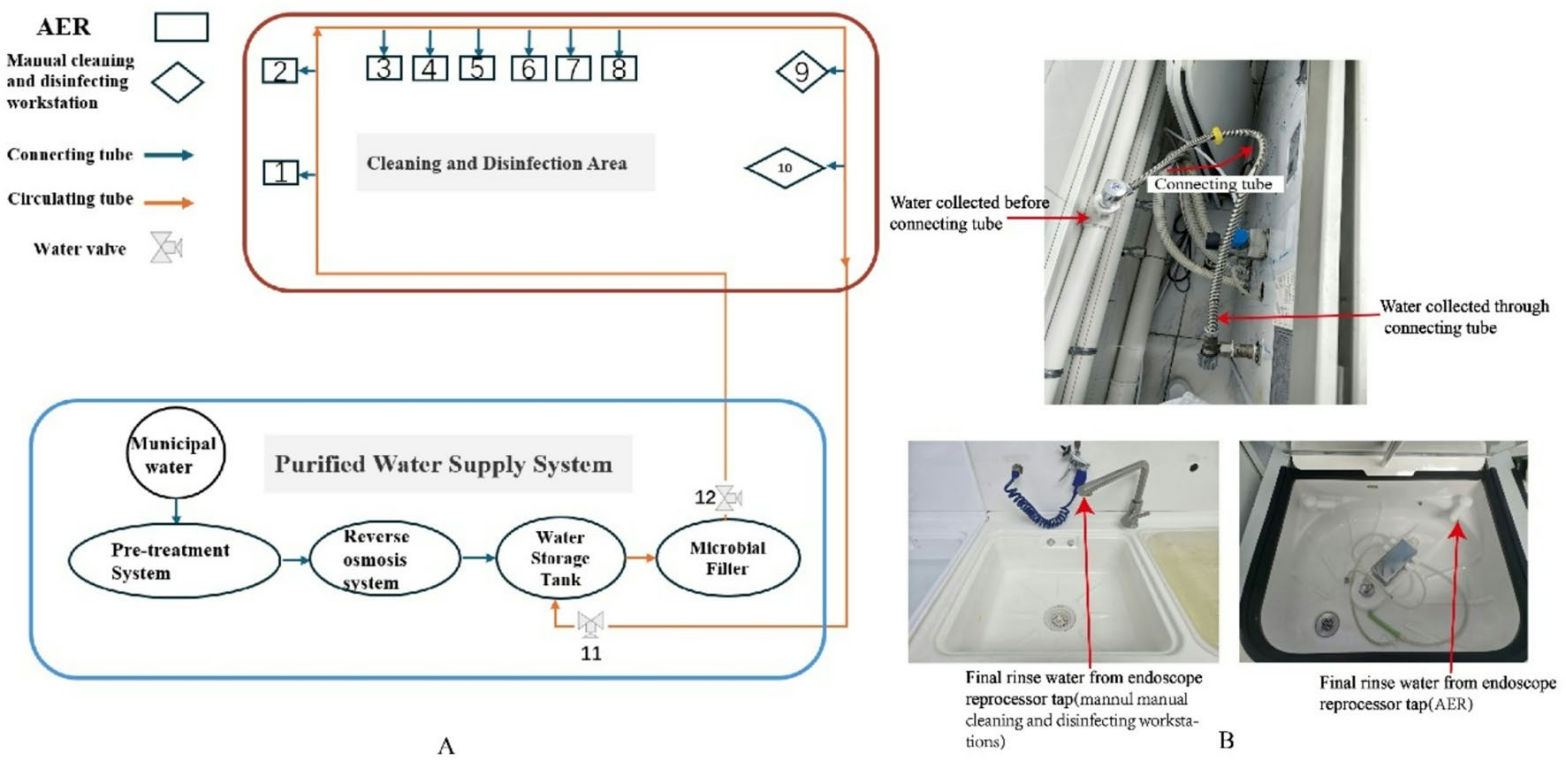
Schematic diagram of sampling locations. (**A**) The diagram of purified water supply system and cleaning and disinfection area. The digestive endoscopy center of our hospital uses a centralized water supply system to meet the center’s demand for purified water. Purified water delivered through the connecting tube to endoscope reprocessors, including 2 manual cleaning and disinfecting workstations and 8 AERs (Automated Endoscope Reprocessor). The number represents the sampling location. (**B**). Schematic diagram of three sampling points of water (Water collected before connecting tube, water collected through connecting tube, final rinse water from endoscope reprocessor tap).

The digestive endoscopy center of our hospital uses a centralized purified water supply system to meet the center’s demand for purified water. The main equipment of the water supply system is located on the top floor of the building. Municipal water is treated through pre-treatment system including sand filtration, carbon filtration, softening, and microfiltration, then purified by reverse osmosis technology. The prepared purified water was disinfected by ozone sterilizer and filtered by microbial filtration and transported to the digestive endoscopy water-use area through a unified circulating tube network (Fig. 1). The total length of the circulating tube network in the Digestive Endoscopy Center is approximately 100 m. Purified water delivered through the connecting tube to equipment who uses purified water, including 2 manual cleaning and disinfecting workstations (Center-R5, Xinhua, Shandong, China) and 8 AERs (Automated Endoscope Reprocessor, Flow-A-200, Xinhua, Shandong, China).

### Flexible GI endoscope reprocessing procedure

In our investigation, inspection of the reprocessing process of flexible GI endoscopes revealed compliance with the guidelines and recommendations, the staff strictly follow the reprocessing steps for flexible endoscopes. The flexible endoscopes underwent leak testing after each use. The outer surfaces were wiped, and the inner channels were brushed with multi-enzyme detergent in manual reprocessing cleaning equipment. The suction button was removed and cleaned. After enzymatic cleaning, the flexible endoscope was placed into the AER which performed enzymatic cleaning, disinfection, final rinsing, alcohol perfusion, and drying. During the final rinsing and drying process, the digestive endoscope channels were flushed with terminal rinse water and dried with 75% alcohol and positive pressure air. Occasionally, the digestive endoscope underwent high-level disinfection, final rinsing, and drying in manual cleaning and disinfecting workstations.

### Study subjects

Endoscopic final rinse water samples and flexible endoscopes were collected. A total of 12 water sample locations were set up, including 8 AERs, 2 manual cleaning and disinfecting workstations and 2 from the outlet and return end of the circulation water system (Fig. 1; Table 1). If the final rinse water is contaminated with waterborne microorganisms, there is a risk of contaminating the endoscope. To explore whether the reprocessing of flexible endoscopes is affected by contaminated final rinse water, we conducted biological surveillance of all 34 reprocessed flexible endoscopes, including 13 colonoscopes, 18 gastroscopes and 3 duodenoscope used in the Fujian medical university union hospital during August 2024. Endoscopes were all reprocessed by AER excluding 3 colonoscopes, which reprocessed in manual cleaning and disinfecting workstations. 102 samples were collected from all channels of 34 endoscopes, including air/water channel, suction/instrument channel and auxiliary water channel of each endoscope. All the disinfection steps were performed, including precleaning, manual cleaning with detergent solution (initial washing), manual or automated high-level disinfection, final rising, drying and storage. Ethics approval was exempted because the study did not involve human subjects or specimens.

**Table 1:** Bacterial detected in the endoscopy purified water before replacing connecting tube.

	Water collected before connecting tube (cfu/100mL)	Water collected through connecting tube (cfu/100mL)	Final rinse water from endoscope reprocessor tap (cfu/100mL)
Location1	0	Uncountable^a^(Chryseobacterium indologenes, Sphingomonas paucimobilis)	Uncountable(Chryseobacterium indologenes,Sphingomonas paucimobilis)
Location2	2*(Sphingomonas paucimobilis)*	Uncountable *(Sphingomonas paucimobilis)*	Uncountable *(Sphingomonas paucimobilis)*
Location3	0	Uncountable *(Chryseobacterium indologenes)*	Uncountable *(Chryseobacterium indologenes)*
Location4	0	Uncountable *(Sphingomonas paucimobilis)*	Uncountable *(Sphingomonas paucimobilis)*
Location5	3*(Sphingomonas paucimobilis)*	Uncountable *(Sphingomonas paucimobilis)*	Uncountable *(Sphingomonas paucimobilis*,* Methylobacterium oryzae)*
Location6	0	75*(Methylobacterium oryzae)*	Uncountable *(Methylobacterium oryzae)*
Location7	0	Uncountable *(Herbaspirillum huttiense*, *Sphingomonas paucimobilis)*	Uncountable *(Methylobacterium oryzae*,* Herbaspirillum huttiense)*
Location8	0	Uncountable *(Methylobacterium oryzae*, *Herbaspirillum huttiense)*	Uncountable *(Methylobacterium oryzae*,* Herbaspirillum huttiense)*
Location9	0	Uncountable *(Chryseobacterium indologenes)*	Uncountable *(Chryseobacterium indologenes)*
Location10	0	Uncountable *(Sphingomonas paucimobilis)*	Uncountable *(Sphingomonas paucimobilis)*
Location11	0	**—** ^**b**^	**—**
Location12	0	**—**	**—**

a. Uncountable means the number of bacteria is too numerous to count.

b. Location11 and 12 collected from water valve from the outlet and end of circulating tube.

### Samples collection and microbiology surveillance

Biological surveillance of water samples: purified water samples were collected from the outlet, end and all points connected to the connecting tube of the circulating tube. Final rinse water samples were collected from manual cleaning and the automatic sterilizer taps. Samples of 100 mL of purified water were collected in sterile containers, followed by laboratory analysis within 2 h. All collected water samples were subjected to bacterial enrichment by filtration through a 0.45 μm membrane. The filter membrane was inoculated on an R_2_A (Reasoner’s 2 A Agar) agar medium and incubated at 30 °C for 5 days. After incubation, the colony count was analyzed, and the threshold for the colony-forming units value was fixed at 10 cfu/100 ml.

Biological surveillance of flexible endoscopes: Biological investigation of endoscopes was conducted. First, 50mL of eluate containing a disinfectant-neutralizing agent was extracted with a sterile syringe and injected into the endoscopic cavity through the biopsy channel, suction channel and auxiliary water channel, followed by air injection. The whole volume was collected in sterile containers within 2 h for testing. All collected specimens were enriched using two 0.45 μm filter membranes. One filter membrane was inoculated on a nutrient agar medium and incubated at 36 °C for 48 h. Following incubation, the colony count was analyzed, with a threshold set at 20 cfu/piece. Strains were identified by MALDI-TOF/MS. Samples were deemed non-compliant if *Enterobacter ales*,* Enterococcus species*,* Staphylococcus species*,* Pseudomonas aeruginosa*, or other non-fermentable gram-negative bacteria were isolated.

### Statistical methods

Statistical analysis was performed using SPSS 19.0 software (IBM, Armonk, NY, USA). Comparison of multiple rates was conducted using either the chi-square test method and the test level was considered significant at *P* ≤ 0.05.

## Result

### Assessment of flexible GI endoscope reprocessing effect and water sample

102 samples collected from 34 endoscopes were tested. No bacterial growth was observed after all the disinfection steps were performed, including brushing, enzymatic cleaning, disinfection, final rinse water flushing, alcohol perfusion and drying, qualification rate was 100% (102/102).

Before the intervention, a total of 32 purified water samples were tested, and the compliance rate was 37.5% (12/32). Only two samples from the outlet and return end of the circulation water tubing (sample location 11 and 12) have no bacterial growth. All 10 samples from final rinse water taps of the 8 automated endoscope reprocessors (AER) (sample location 1–8) and 2 manual cleaning and disinfecting workstations (sample location 9–10) exceeded the normal standard. Except for the water sample from AER 6, which had a bacterial count of 75 cfu/100mL, all other samples contained too numerous bacteria and could not be counted.

Four types of gram-negative bacteria have been discovered in 22 positive samples, containing *Chryseobacterium indologenes*, *Sphingomonas paucimobilis*,* Methylobacterium oryzae* and *Herbaspirillum huttiense.* The dominant bacteria is *Sphingomonas paucimobilis*, which was found in 14 samples, with a composition ratio is 43.75% (14/32). Others are *Methylobacterium oryzae* (8/32, 25%), *Chryseobacterium indologenes* (6/32, 18.75%), and *Herbaspirillum huttiense* (2/32, 6.25%).

Then, the engineer replaced the 10 connecting tubes (the tube connecting the circulating tube and the endoscope reprocessing equipment). The length of the connecting tube is between 85 cm and 240 cm. We found that the inner surface of each replaced connecting tube was rough, with heavy soiling and residual purified water. Innumerable OPPPs were found in all 10 residual water samples, and the incompliance rate was 100% (10/10). After replacing the connecting tube, we conducted monitoring again at the same 32 sampling points. All water samples meet standards; the compliance rate developed to 100% (32/32) (*P* = 6.9 × 10^− 8^). A small number of bacteria grown at No. 5 to No. 7 AER and their connecting tube (Table 2). The dominant bacteria is *Methylobacterium oryzae*, which was found in 7 samples, with a relative abundance of 21.88% (7/32). The other is *Sphingomonas paucimobilis*, which was found in 2 samples, with a relative abundance of 9.38% (3/32).

**Table 2:** Bacterial detected in the endoscopy purified water after replacing connecting tube.

	Water collected before connecting tube (cfu/100mL)	Water collected through connecting tube (cfu/100mL)	Final rinse water from endoscope reprocessor tap (cfu/100mL)
Location1	0	0	0
Location2	0	0	0
Location3	0	0	0
Location4	0	0	0
Location5	3*(Sphingomonas paucimobilis)*	7(*Sphingomonas paucimobilis)*	5*(Sphingomonas paucimobilis)*
Location6	1*(Methylobacterium oryzae)*	5*(Methylobacterium oryzae)*	4*(Methylobacterium oryzae)*
Location7	0	6*(Methylobacterium oryzae)*	6*(Methylobacterium oryzae)*
Location8	0	1*(Methylobacterium oryzae)*	4*(Methylobacterium oryzae)*
Location9	0	0	0
Location10	0	0	0
Location11	0	**—** ^a^	**—**
Location12	0	**—**	**—**

a. Location11 and 12 collected from water valve from the outlet and end of circulating tube.

## Discussion

In this study, we investigated a cluster of non-compliant purified water results detected during routine quarterly microbiological surveillance in the Digestive Endoscopy Center in August 2024. The colony count of purified water used for final rinse of endoscopes after disinfection protocols from endoscope cleaning and disinfection equipment exceeded the standard during routine microbiologic monitoring in Center for Digestive Endoscopy. The purified water samples remained unqualified until the connecting tubes were completely replaced.

Previous studies have established that contamination of final rinse water could cause post-endoscopic infections, with *Pseudomonas aeruginosa* and *Legionella pneumophila* being the most commonly implicated pathogens^7–11^. These microorganisms belong to the group known as Opportunistic Premise Plumbing Pathogens (OPPPs), which are normal inhabitants of natural and distributed water systems^5,6^. OPPPs possess adaptive traits—such as growth under oligotrophic conditions and persistence in stagnant water—that enable them to colonize plumbing networks^12^. Notably, an outbreak of *P. aeruginosa* infections in ICU patients has been directly linked to contaminated bronchoscope rinse water. The authors isolated *Pseudomonas aeruginosa* from sputum specimens obtained from patients, as well as from bronchoscopes and the final rinse water^13^. Consistently, the organisms recovered from our final rinse water samples (*Sphingomonas paucimobilis*, *Herbaspirillum huttiense*, and *Chryseobacterium indologenes*) are also recognized OPPPs capable of causing nosocomial infections^14,15^. Patients who underwent endoscope procedures were at risk of acquiring OPPPs. Fortunately, no instances of flexible endoscope reprocessing failure were attributed to contamination of final rinse water, as we monitored bacteria from all channels of 34 endoscopes used during August. We conducted telephone follow-ups with patients who underwent endoscopic procedures in August and found no related infection cases. This may be because after the final flushing by purified water, the endoscope still requires an additional 2-minute flush with 75% ethanol and drying with compressed air before it can be used for clinical procedures. Furthermore, for high-risk endoscopes like duodenoscopes, we perform formaldehyde fumigation sterilization after endoscope reprocessed. Our experience aligns with studies demonstrating that thorough drying, facilitated by alcohol, can serve as an effective barrier against bacterial proliferation^16^, and properly dried endoscopes have not been directly linked to nosocomial infections^17^. One study has suggested that flushing 70% ethanol through endoscope channels followed by drying with compressed air could effectively prevent *P aeruginosa* contaminations^18^. Major US guidelines recommend rinsing the endoscope channels with purified water after disinfection, followed by flushing the channels with 70%-90% ethanol or isopropyl alcohol^19^. However, this incident should still be regarded as a “near-miss”, exposing a critical vulnerability in current guidelines. While alcohol provides drying assistance and bactericidal effects, current studies have not clearly investigated the relationship between the degree of final rinse water contamination, alcohol perfusion and drying progress, and the contamination of flexible endoscopes, it depends on the future studies. The absence of patient infections resulted from successful interception at the drying step, not absence of risk. This near-miss highlights potential over-reliance on terminal drying to compensate for upstream failures, underscoring the need for guidelines to enforce stricter control over the entire water delivery infrastructure, rather than solely depending on post-rinse interventions.

For our purified water supply system, routine daily disinfection of purified water was performed with ozone sterilizer in the purified water tank and 0.22 μm filtering membrane placed in the faucet where the purified water discharges from purified water tank to the circulating tube (Fig. 1). According to product manual, filtering membrane was replaced every 6 months by third-party companies and hospital equipment affairs, and ozone sterilizer was replaced every year. In this study, no microbial growth was found in purified water samples before connecting tube, suggesting that no contamination of OPPPs in circulating tube. However, OPPPs were found on final rinse water cultured from tap of endoscope reprocessed equipment. During the revised process, neither filtering membrane nor ozone sterilizer replacement had any effect on elimination of OPPPs. After replacement of connecting tubes between circulating tube and endoscope reprocessing equipment, successful eradication of OPPPs was observed.

Hospital water supply pipelines provide a nearly ideal environment for microbial survival and proliferation^20^. Within the protective matrix of biofilms, bacteria can resist heat, disinfectants, and even antibiotics, contributing to persistently high non-compliance rates in medical water quality. In China, a multicenter study reported that the compliance rates of final rinse water for endoscopes were only 63.09% (53/84) and 61.11% (111/180), respectively^20^. Studies have shown that the primary causes of contamination in final rinse water is the failure to regularly replace the filter^10,21,22^, improper filter pore size^23^, filter missing^24^ and ineffective disinfection of water supply pipelines^20^. This is, however, inconsistent with the findings of this survey. In this study, we found that there is a section of long connecting tube between the purified water circulating tube and the endoscope reprocessed equipment, to ensure purified water from circulating tube to reprocessed equipment. Water in this section of the tube cannot return to the circulating tube. To support the digestive endoscopy center’s clinical operations, the endoscope reprocessed equipment in the endoscope reprocessing center is in operation from 7:30 AM to 6:00 PM on weekdays (Monday through Friday). This provides a favorable opportunity for water retention within the connecting tubes, leading to bacterial growth. However, current international and domestic guidelines primarily focus on bacterial counts in water quality, lacking strict and comprehensive requirements for pipeline maintenance measures. We also did not find any maintenance strategies for the connecting tube in the product manual of endoscope reprocessor and purified water supply system. Thus, daily cleaning, disinfection, and maintenance of connecting tubes are neglected. Furthermore, we discovered the 10 suspected connecting tubes that were replaced featured a double-layered structure, with the outer layer consisting of corrugated tubing made of stainless steel and the inner layer made of EPDM (Ethylene Propylene Diene Monomer). According to recent study, EPDM exhibits high potential for biofilm formation among commonly used pipe materials. In a comparative study of six common pipe materials (PP, PVC, EPDM, PEX, SS, Cu), the density of biofilm formed on material surfaces ranked as follows: EPDM > PP (Polypropylene) > PVC (Polyvinyl Chloride) > PEX (Cross-linked Polyethylene) > Cu (Copper) – SS (Stainless Steel). This indicates that EPDM is more prone to supporting biofilm growth when compared to metallic materials such as stainless steel and copper^25^. Therefore, we selected tubing made entirely of type 316 stainless steel as the new connecting tubes, with connectors at both ends also made of stainless steel, to meet the requirements for transporting final rinse water with extremely high cleanliness standards. Because of the cost of the implementation, regular replacement of the connecting tubes is not very practical. We recommend disassembling the connecting tubes every week and disinfecting them by immersing them in chlorine-containing disinfectant at a concentration of 500 mg/L for 30 min. In the following year, the monitoring results of final rinse water showed that the compliance rate remained consistently high (all ≥ 90%). This suggested that weekly disinfection of the connecting tubes can effectively control the growth of OPPPs. In the future, we plan to redesign the purified water supply pipeline layout, shortening the distance between the circulation pipeline and the endoscope reprocessor. It can help shorten the connecting tube to prone to stagnant water. However, further research and discussion are needed to determine its applicability.

To prevent bacterial growth or biofilm in purified water transport, appropriate measures are needed. The installation of membrane filters at the terminal of water for final rinse is a critical step in reducing the number of bacteria in rinse water^20^. Furthermore, the main purified water disinfection methods including chemical disinfection, ultraviolet disinfection, and ozone disinfection were often used in combination with the filtering membrane method, significantly enhance the compliance rate of microbial detection in the final rinse water by not only producing qualified final rinse water but also disinfecting the water tube^13,20^. In this study, ozone disinfection comminated with filtering membrane was used for eliminating bacteria in purified water. However, OPPPs cultured from filtered water suggested that the effect of 0.22 μm filtering membrane was limited on account of the degree of contaminated water requiring filtration. The correlation between the degree of contaminated water filtered and its effect on membrane filtering will be studied in our further research.

In this study, we report OPPPs contamination in final rinse water in digestive endoscopy center, for which connecting tube between circulating tube and endoscope reprocessors was the hidden reservoir for contaminating final rinse water. The results highlight the importance of routine biological surveillance and thorough cleaning of the nondetachable connecting tube in order to control the quality of endoscope cleaning and disinfection.

## Data Availability

All data generated or analysed during this study are included in this published article.
